# Parents’ intention to vaccinate their 5- to 11-year-old children with the COVID-19 vaccine: rates, predictors and the role of incentives

**DOI:** 10.1186/s12889-023-15203-y

**Published:** 2023-02-14

**Authors:** Liora Shmueli

**Affiliations:** Department of Management, Bar-Ilan University, 52900 Ramat-Gan, Israel

**Keywords:** Health belief model, Incentives, SARS-CoV-2, Vaccine acceptance

## Abstract

**Introduction:**

Immediately after Pfizer announced encouraging effectiveness and safety results from their COVID-19 vaccine clinical trials in 5- to 11-year-old children, this study aimed to assess parents’ perceptions and intention to vaccinate their 5-11-year-old children and to determine socio-demographic, health-related, behavioral factors, as well as the role of incentives.

**Methods:**

A cross-sectional online survey of parents of children between 5 and 11 years of age among the Jewish population in Israel (n = 1,012). The survey was carried out between September 23 and October 4, 2021, at a critical time, immediately after Pfizer’s announcement. Two multivariate regressions were performed to determine predictors of parents’ intention to vaccinate their 5-11-year-old children against COVID-19.

**Results:**

Overall, 57% of the participants reported that they intend to vaccinate their 5-11-year-old children against COVID-19 in the winter of 2022. 27% noted that they would vaccinate their 5-11-year-old children immediately; 26% within three months; and 24% within more than three months. Perceived susceptibility, benefits, barriers and cues to action, as well as two incentives - vaccine availability and receiving a “Green Pass” - were all significant predictors. However, Incentives such as monetary rewards or monetary penalties did not increase the probability of parents’ intention to vaccinate their children. Parental concerns centered around the safety of the vaccine, fear of severe side effects, and fear that clinical trials and the authorization process were carried out too quickly.

**Conclusion:**

This study provides data on the role of incentives in vaccinating 5-11-year-old children, how soon they intend to do so, and the predictors of those intentions, which is essential knowledge for health policy makers planning vaccination campaigns.

**Supplementary Information:**

The online version contains supplementary material available at 10.1186/s12889-023-15203-y.

## Background

The heavy toll the COVID-19 pandemic has levied on children is becoming increasingly apparent. According to a recent national report, children in the US now account for more than one-fourth of COVID-19 weekly reported cases, 1.6-4.2% of the total cumulative COVID-19 hospitalizations, and 0.00-0.25% of all COVID-19 deaths (depending on the reporting state) [1]. Many infected children (both symptomatic and asymptomatic) are also experiencing long-term effects, that may last for many months after the initial infection, with some studies reporting rates higher than 50% [2]. Beyond the direct health-related outcomes of an infection, the COVID-19 pandemic has affected children in many other manners, including psychologically, educationally and economically [3]. Implementing a preventive measure, such as vaccination, to reduce infection among children is, therefore, critical to mitigate the severe outcomes and long-term effects in children, as well as to increase community protection.

At the time of conducting this study (October 2021), several vaccines against COVID-19 were authorized by the U.S. Food and Drug Administration agency (FDA). Among them is the Pfizer-BioNTech vaccine, whose approved use in individuals aged > 16 years in December 2020 [4] has since been extend to include children above 12 years of age [5]. While, at that time, vaccines for younger children have not yet been authorized, on September, 20, 2021, Pfizer announced encouraging efficacy and safety results from its clinical trials of children aged 5 to 11 years [6]. However, even when authorized, these vaccines can only be effective if parents are in favor of vaccinating their kids.

Despite the availability of COVID-19 vaccines for adults and adolescents, not all eligible individuals have chosen to get vaccinated, in part due to a phenomenon known as vaccine hesitancy. COVID-19 vaccine hesitancy among adults has been well-studied. Most of the concerns in this context relate to the safety of the vaccines (e.g., their long-term side effects, the speed in which they were developed and authorized, etc.) and their effectiveness (e.g., how long immunity would last, effectiveness against new variants, etc.) [7–10]. However, little is known about parental hesitancy regarding vaccinating their offspring against COVID-19, and in particular for those 5–11 years of age.

To actively promote voluntary COVID-19 vaccinations among adults and adolescents, several incentive-based strategies have been proposed. One such incentive is monetary rewards. In the USA, for example, John Delaney and Robert Litan proposed paying people $US 1000–1500 to get vaccinated [11, 12]. Another strategy, suggested by several governments, including those of Chile, Germany, Italy, the UK, and the USA, is the use of “immunity passports” and “vaccination certificates” [13]. Another non-monetary incentive model developed by the Israeli Ministry of Health termed the “Green Pass”. Holders of a Green Pass are permitted to enter facilities and social events, such as hotels, restaurants and concerts; they were only issued to those who recovered from COVID-19 or are fully vaccinated [12, 14]. Among the various strategies explored, only vaccine availability was shown to increase the likelihood of getting vaccinated immediately [15], whereas incentives such as a monetary reward or monetary penalty (e.g., reducing social security benefits) or a Green Pass were not found to be significant predictors of getting vaccinated among the adult population [15]. However, to the best of our knowledge, no study thus far has examined the association between such incentives and parents’ willingness to vaccinate their young children.

As such, the goal of this paper is twofold. First, it aims at assessing parents’ intention to vaccinate their 5-11-year-old children in the winter of 2022, before the approval of the COVID-19 vaccine for this age group, and how soon they intend to do so. Second, it aims at determining multiple factors associated with predicting these intentions. Specifically, it examines: (1) socio-demographic factors, such as age group, gender, and level of education; (2) health-related factors, such as whether the respondent got vaccinated against COVID-19 and having a family member suffering from a chronic disease; (3) behavioral factors based on the Health Belief Model (HBM)^1^, such as perceived susceptibility, perceived severity, and perceived benefits; and (4) incentive-related factors, such as vaccine availability, and monetary rewards.

## Methods

### Study participants and survey design

A cross-sectional online survey of parents of children aged 5–11 years was conducted among the Jewish population in Israel. The survey was carried out between September 23 and October 4, 2021, immediately after Pfizer announced its results of a vaccine clinical trial in children aged 5 to 11 years. The Pfizer-BioNTech’s COVID-19 vaccine was reported to have a favorable safety profile and to generate a robust immune response.

Our survey was distributed by Sarid Research Institute for Research Services via an online panel containing a wide pool of potential interviewees who had consented to participate in surveys from time to time. Eligible participants were required to be (1) 18 + years old and (2) parents of a child aged 5 to 11 years. To compose a representative sample of the Jewish adult population in Israel, respondents were sampled by layers based on age, gender, geographic area, and level of religiosity.

The questionnaire used in the current study is partly based on a previous questionnaire distributed to the general public in June 2020, which was prepared after consultation with a panel of public health experts, including a statistician, a behavioral psychologist and an epidemiologist [19]. After adjusting the questionnaire to the goals of the current study, it was refined by receiving feedback from parents of young children regarding its comprehensibility, usability and time taken to complete the survey. Then, the questionnaire was pre-tested on a relatively small group of 100 respondents. After the reliability of the questionnaire was verified using a Cronbach α internal reliability test (the HBM section of the questionnaire obtained an internal consistency of Cronbach α = 0.75) it was distributed to the rest of the respondents.

### Questionnaire

The questionnaire (Supplementary Material 1-Supplementary Questionnaire 1) consisted of the following sections: (1) Socio-demographic predictor variables; (2) health-related predictor variables; (3) HBM predictor variables and (4) incentive-related predictor variables that could accelerate parents’ inclination to vaccinate their children. Overall, the questionnaire consisted of 40 questions, all of which were mandatory.

### Variables and measurements

The first dependent variable was parents’ intention to vaccinate their 5-11-year-old children against COVID-19 in the winter of 2022, measured as a one-item question on a 1–6 scale (1 - strongly disagree; 6 - strongly agree). The second dependent variable was the sense of urgency of parents to vaccinate their 5-11-year-old children against COVID-19, split into 3 categories of vaccination intent: immediately, within 3 months, and more than 3 months^2^ (responded that would not vaccinate their children at all were not included in the sense of urgency to get vaccinated analysis).

Independent variables were grouped into four blocks:

1. Socio-demographic predictor variables: (1) age group; (2) gender; (3) level of education; (4) marital status; (5) socio-economic level^3^; (6) periphery level, defined by the residential area^4^; (7) religiosity level (secular, traditional, religious, orthodox); and (8) working as a medical practitioner. The age variable was transformed from a numeric to a categorical one (18–39; 40+) in order to address differences between specific age groups^5^.
2. Health-related predictor variables: (1) whether the respondent got vaccinated against COVID-19; (2) having a family member suffering from a chronic disease (one or more of the following: heart disease; vascular disease and/or stroke; diabetes mellitus; hypertension; chronic lung disease, including asthma or immune suppression); (3) the existence of past episodes of COVID-19 in the family; (4) the existence of past episodes of hospitalization in the family in the previous year; and (5) whether the children of the respondent got vaccinated against influenza in the previous year.
3. HBM predictor variables: (1) perceived susceptibility (included two items); (2) perceived severity (included two items); (3) perceived benefits (included three items); (4) perceived barriers (included two items); (5) cues to action (included three items); and (6) health motivation (included one item). The HBM items were measured on a 1–6 scale (1 - strongly disagree; 6 - strongly agree). Negative items were reverse scored. Scores for each item were averaged to obtain each of the HBM-independent categories. The Cronbach α internal reliability method revealed the internal consistency of the HBM section to be Cronbach α = 0.79 (Supplementary Table 1 in Supplementary Material 2).
4. Incentive-related predictor variables: (1) vaccine availability (“If the vaccine would become accessible and available at school”); (2) monetary rewards; (3) receiving a Green Pass (“If my child would receive a Green Pass that would grant various restriction exemptions (exemption from isolation, entry to places of entertainment etc.)”); and (4) monetary penalties (“If the government would reduce my social security benefits or impose another fine in the case that my child does not get vaccinated”).

### Statistical analyses

Data from the online questionnaires were analyzed using SPSS 26 software, where explicit identifiers were replaced by coded pseudo-identifiers. To test the reliability of HBM measures, the Cronbach’s α test was used. To describe characteristics of the study population, the following methods of descriptive statistics were used: frequencies, percentages, averages, and standard deviations.

Relationships between dependent and independent variables were examined by univariate analysis, using either ANOVA or t-tests on independent samples or Chi-squared tests, depending on the characteristics of the variable examined. Cohen’s d and Eta squared were used to measure the effect size for significant effects.

Two multivariate regressions were performed. First, to investigate determinants of parents’ intention to vaccinate their children against COVID-19 in the winter of 2022, a four-step hierarchical binary logistic regression was performed^6^. The intention to vaccinate children served as the dependent variable. In the initial step, only the socio-demographic variables found to be significant (p < .05) in the univariate analysis were inserted into the regression model as predictors. In the second step, the health-related variables found to be significant (p < .05) in the univariate analysis were entered as predictors. In the third step, all the HBM variables were entered into the model as predictors. In the fourth and final step, four incentive-related variables were entered as predictors (vaccine availability, monetary rewards, monetary penalties and receiving a Green Pass).

Second, to estimate the sense of urgency of parents to vaccinate their children against COVID-19, a multinomial logistic regression was estimated. Specifically, I was interested in predicting what would increase the intention to vaccinate of parents who stated that they would vaccinate their children within 3 months or more than 3 months from the moment the vaccine becomes available, compared to those who opted for the immediate possibility. Only socio-demographic and health-related variables that were found to be significant (p < .05) in the univariate analysis were inserted into the regression model as predictors. All the HBM and incentive-related variables were entered into the model as predictors.

## Results

### Participant characteristics

Overall, 1,012 parents of children aged 5–11 years completed the survey^7^; 51% of them were female (n = 516). Almost half the parents (n = 489) were 18–39 years old. The participants were distributed nearly equally among the three socio-economic categories (low, medium, and high), with 13% of the respondents (n = 135) living in peripheral geographical regions. Almost half the respondents (n = 498) were secular, 60% (n = 625) held an academic degree, most (89%, n = 902) were married, and 23% (n = 231) stated having a family member suffering from a chronic disease. A comparison of the sample and the entire population in terms of the characteristics used for sampling: age, gender, level of religiosity, and geographical area, is provided in Supplementary Table 2 in Supplementary Material 2. Among respondents with children aged 12–15 years (42%, n = 422), just over half (n = 214) stated these children were vaccinated against COVID-19. The descriptive characteristics of the respondents are provided in Supplementary Table 3 in Supplementary Material 2.

### Rates of intention to vaccinate children

When parents were asked regarding their intention to vaccinate their 5-11-year-old children against COVID-19 in the winter of 2022, 57% of them (n = 579) stated that they intend to vaccinate their 5-11-year- old children against COVID-19 in the winter of 2022. When asked, how soon they intend to vaccinate their children, 27% of the parents (n = 270) responded that they intend to vaccinate their children immediately, within less than one month; 26% (n = 267) answered that within one-to-three months; and 24% (n = 242) stated that they would wait longer: 17% within 4-to-12 months and 7% would wait more than a year. The remainder, 23% (n = 233), responded that they would not vaccinate their children in this age group at all, and as such were not included in the sense of urgency to get vaccinated analysis.

### Main reasons for the reluctancy to vaccinate children

The main reasons that discouraged parents from vaccinating their 5-11-year-old children immediately (n = 742) were: concerns about the safety of the vaccine for children (64%), fear of severe side effects of the vaccine (60%), and fear that the vaccine clinical trials and the authorization process were carried out too quickly (56%). Additional reasons were the impression that COVID-19 is not dangerous for children in this age group, so there is no reason to vaccinate them (33%), and concerns regarding low vaccine efficacy (25%).

### Univariate analyses: intention to vaccinate 5-11-year-old children

The results of the univariate analyses between socio-demographic and health-related variables and parents’ intent to vaccinate their 5-11-year-old children against COVID-19 in the winter of 2022 are reported in Supplementary Table 3 in Supplementary Material 2. The findings show that men are more likely to vaccinate their offspring than women (63% vs. 52%, p < .001, respectively), as are parents over the age of 40 compared to younger ones (64% vs. 49%, p < .001, respectively), those with an academic degree compared to those without one (60% vs. 53%, p < .05, respectively), and individuals with a higher-than-average income compared to those with a below-average and average income (67% vs. 52%, p < .001, respectively). Interestingly, parents whose children received the flu vaccine in the winter of 2021 expressed a significantly higher willingness to vaccinate their children against COVID-19 in the winter of 2022 than those whose children did not receive a flu vaccine in the previous winter (67% vs. 49%, p < .001, respectively). Parents who were vaccinated against COVID-19 conveyed greater readiness to vaccinate their 5-11-year-old children compared to those who were not vaccinated (61% vs. 15%, p < .001, respectively). Among the parents who also had children aged 12–15 years (n = 422), those whose 12-15-year-old children were vaccinated against COVID-19 expressed a stronger intention to vaccinate their 5-11-year-old children, compared to those whose 12-15-year-old children were not vaccinated (77% vs. 46%, p < .001, respectively). No significant differences were found for the other demographic variables assessed: religious denomination, marital status, working as a medical practitioner, periphery level, having a family member suffering from a chronic disease, past episodes of COVID-19 in the family, and past episodes of hospitalization in the family in the previous year.

The results of the univariate analyses between the HBM and incentive-related variables and the intention to vaccinate 5-11-year-old children against COVID-19 in the winter of 2022 are reported in Supplementary Table 4 in Supplementary Material 2. Perceived susceptibility (p < .01, Cohen’s *d =* 2.08), perceived benefits (p < .01, Cohen’s *d =* 1.83) perceived barriers (p < .01, Cohen’s *d =* 1.19), cues to action (p < .01, Cohen’s *d =* 1.52) and health motivation (p < .01, Cohen’s *d =* 0.43) all had a statistically significant effect on parent’s intention to vaccinate their offspring. No significant effect was found for perceived severity. With respect to incentive-related variables, all four incentives: availability (p < .01, Cohen’s *d =* 2.00), monetary rewards (p < .01, Cohen’s *d =* 1.24), monetary penalties (p < .01, Cohen’s *d =* 1.09) and Green Pass (p < .01, Cohen’s *d =* 1.83) were found to have statistically significant effects on the intention of parents to vaccinate their 5-11-year-old children against COVID-19 in the winter of 2022. Notably, the effect sizes in all of these cases were relatively large.

### Univariate analyses: sense of urgency to vaccinate 5-11-year-old children

The results of the univariate analyses between the socio-demographic and health-related variables and the sense of urgency to vaccinate 5-11-year-old children against COVID-19 are reported in Supplementary Table 5 in Supplementary Material 2. The predictor variables found to have a statistically significant effect (p < .05) on the sense of urgency are gender, socio-economic level, having a family member suffering from a chronic disease, and whether their children were vaccinated against influenza in the previous winter. The predictor variables not found to have a statistically significant effect include age group, educational level, marital status, periphery level, religiosity level, past episodes of COVID-19 in the family, and past episodes of hospitalization in the family in the previous year.

The results of the univariate analyses between the HBM variables and incentive-related variables and the sense of urgency to vaccinate 5-11-year-old children against COVID-19 are reported in Supplementary Table 6 in Supplementary Material 2. The results in this case are consistent in terms of statistical significance with those reported in Supplementary Table 4 in Supplementary Material 2 for the case of the intention to vaccinate 5-11-year-old children against COVID-19 in the winter of 2022. However, their effect sizes were relatively smaller.

### Multivariate analyses: predictors of the intention to vaccinate 5-11-year-old children

The first regression analysis accounted for an estimated 80% of the explained variance in the intention to vaccinate 5-11-year-old children against COVID-19 in the winter of 2022 (Naglekerke’s pseudo *R*^*2*^ = 0.80). All the model’s steps were significant. The most important components of the hierarchical regression were the HBM dimensions, which added 61% to the estimate of the explained variance, on top of the 17% explained by socio-demographic and health-related characteristics. The four incentives added 2% beyond those offered by HBM.

More specifically, according to the final model, among the socio-demographic and health-related variables, only age group was found to be significantly associated with the intention to vaccinate 5-11-year-old children against COVID-19 in the winter of 2022, where parents over the age of 40 were 2.45 times more likely to vaccinate their children than parents below the age of 40 (medium effect size - OR = 2.45, 95% CI 1.50–4.02). No other socio-demographic or health-related variable was found to be a significant predictor.

Among the HBM variables, perceived susceptibility (medium effect size - OR = 2.70, 95% CI 1.97–3.69), perceived benefits (medium effect size - OR = 2.54, 95% CI 1.74–3.69) and cues to action (very small effect size - OR = 1.44, 95% CI 1.07–1.94), were all significant positive predictors of the intention to vaccinate 5-11-year-old children against COVID-19 in the winter of 2022. In contrast, perceived barriers (small effect size - OR = 0.53, 95% CI.41–0.69) was found to be a negative significant predictor. Perceived severity and health motivation were not found to be significant predictors.

Among the incentive-related variables, only vaccine availability (small effect size - OR = 1.69, 95% CI 1.30–2.20) and receiving a Green Pass (very small effect size - OR = 1.32, 95% CI 1.04–1.67) were found to be significant positive predictors of the intention to vaccinate 5-11-year-old children against COVID-19 in the winter of 2022. Monetary rewards and monetary penalties were not found to be significant predictors.A complete description of all the model’s steps, goodness of fit indices and regression coefficients are provided in Table 1.

**Table 1:** Hierarchical logistic regression analysis - predictors of parents’ intention to vaccinate their children aged 5–11 years against COVID-19 (n = 1,012)

	Model 1		Model 2		Model 3		Model 4	
Variable	*b(se)*	*OR [95% CI [*	*b(se)*	*OR [95% CI]*	*b(se)*	*OR [95% CI]*	*b(se)*	*OR [95% CI]*
**Socio-demographic**								
Age Group (40+)	0.54***(0.13)	1.72[1.33,2.23]	0.51***(0.14)	1.67[1.27,2.18]	0.91***(0.24)	2.49[0.1.56,3.97]	0.90***(0.25)	2.45[1.50,4.02]
Gender (Female)	− 0.37**(0.13)	0.69[0.54,0.90]	− 0.45***(0.14)	0.64[0.49,0.84]	− 0.19(0.24)	0.83[0.52,1.31]	− 0.03(0.25)	0.97[0.59,1.59]
Education (Academic)	0.17(0.14)	1.19[0.90,1.56]	0.06(0.15)	1.06[0.79,1.42]	0.14(0.25)	1.15[0.71,1.86]	0.06(0.26)	1.06[0.64,1.77]
Low socioeconomic status	0.10(0.16)	1.10[0.81,1.51]	0.11(0.17)	1.12[0.80,1.56]	0.03(0.28)	1.03[0.60,1.77]	0.09(0.29)	1.10[0.62,1.95]
High socioeconomic status	0.52**(0.16)	1.68[1.22,2.31]	0.45**(0.17)	1.56[1.12,2.18]	0.12(0.29)	1.13[0.64,1.99]	0.17(0.31)	1.18[0.65,2.16]
**Health-related**								
Vaccine parent (COVID-19)			2.01***(0.32)	7.46[4.02,13.8]	− 0.71(0.58)	0.49[0.16,1.52]	-1.17(0.62)	0.31[0.09,1.04]
Vaccine child (Flu)			0.66***(0.14)	1.94[1.48,2.55]	0.22(0.24)	1.25[0.78,1.99]	0.29(0.25)	1.33[0.81,2.18]
**HBM**								
Perceived Susceptibility					1.08***(0.15)	2.95[2.18,3.98]	0.99***(0.16)	2.70[1.97,3.69]
Perceived Severity					0.21(0.14)	1.24[0.94,1.62]	0.17(0.15)	1.19[0.89,1.58]
Perceived Benefits					1.09***(0.18)	2.99[2.10,4.25]	0.93***(0.19)	2.54[1.74,3.69]
Perceived Barriers					− 0.77***(0.13)	0.46[0.36,0.60]	− 0.63***(0.13)	0.53[0.41,0.69]
Cues to action					0.78***(0.13)	2.19[1.71,2.81]	0.36*(0.15)	1.44[1.07,1.94]
Health motivation					− 0.15(0.12)	0.86[0.69,1.09]	− 0.11(0.12)	0.89[0.70,1.14]
**Incentives**								
Availability							0.52***(0.14)	1.69[1.30,2.20]
Monetary reward							− 0.02(0.11)	0.98[0.79,1.21]
Green pass							0.28*(0.12)	1.32[1.04,1.67]
Monetary penalty							− 0.08(0.10)	0.93[0.77,1.12]
Constant	− 0.09(0.17)	0.91	-2.10***(0.34)	0.12	-7.83**(1.31)	0.00	-8.09***(1.32)	0.00
Model${\chi }^{2}$	48.139,p < .001		138.53,p < .001		872.64,p < .001		915.94,p < .001	
Step${\chi }^{2}$	48.139,p < .001		90.39, p < .001		734.11, p < .001		42.30, p < .001	
${\text{C}\text{o}\text{x} \& \text{S}\text{n}\text{e}\text{l}\text{l} R}^{2}$	0.05		0.13		0.58		0.60	
${\text{N}\text{a}\text{g}\text{l}\text{e}\text{k}\text{e}\text{r}\text{k}\text{e} R}^{2}$	0.06		0.17		0.78		0.80	

*p < .05, **p < .01, ***p < .001, (n = 1012)

For robustness, an alternative analysis was conducted in which the intention to vaccinate variable was analyzed in its original scale (without transformation). This alternative analysis is reported in Supplementary Analysis 3.

### Multivariate analyses: predictors of the sense of urgency to vaccinate 5-11-year-old children

The second regression analyzed the sense of urgency of parents to vaccinate their 5-11-year-old children against COVID-19 once the vaccine is approved for this age group. I was specifically interested in predicting what would increase the intention to vaccinate children in this age group immediately, rather than within 3 months or within more than 3 months from the time the vaccine becomes available. The resulting model was found to be significant.

Namely, none of the socio-demographic variables were found to be significantly associated with the sense of urgency to vaccinate 5-11-year-old children against COVID-19. As to the health-related variables, two were found to be significant predictors. Specifically, parents were less inclined to vaccinate immediately, but preferred to vaccinate within 3 months, if they weren’t having a family member suffering from a chronic disease (medium effect size - OR = 0.57, 95% CI.35-0.93). Parent were less inclined to vaccinate immediately but preferred to vaccinate within 3 months or within more than 3 months, if their children weren’t vaccinated against influenza in the previous winter (medium effect size - OR = 0.60, 95% CI.39-0.91, medium effect size - OR = 0.53, 95% CI.30-0.92 respectively).

With the exception of health motivation, all the HBM tested variables were found to be significant predictors of the sense of urgency to vaccinate 5-11-year-old children against COVID-19. Specifically, for each unit increase in perceived susceptibility, the intention to vaccinate in 3 months rather than immediately decreases 0.64-fold (small effect size - OR = 0.64, 95% CI 0.47-0.86), while the intention to vaccinate within a year rather than immediately decreases 0.37-fold (medium effect size - OR = 0.37, 95% CI.25-0.55).

For each unit increase in perceived severity, the intention to vaccinate in 3 months rather than immediately doesn’t significantly change (OR = 0.94, 95% CI 0.76-1.18). Yet, the intention to vaccinate within a year rather than immediately decreases 0.70-fold (very small effect size - OR = 0.70, 95% CI.51-0.96). Furthermore, for each unit increase in perceived benefits, the intention to vaccinate in 3 months rather than immediately decreases 0.62-fold (small effect size - OR = 0.62, 95% CI.44-0.89), while the intention to vaccinate within a year rather than immediately decreases 0.43-fold (medium effect size - OR = 0.43, 95% CI.27-0.67). Conversely, for each unit increase in perceived barriers, the intention to vaccinate in 3 months rather than immediately increases 1.62-fold (small effect size- OR = 1.62, 95% CI 1.30–2.02), while the intention to vaccinate within a year rather than immediately increases 2.62-fold (medium effect size - OR = 2.62, 95% CI 1.92–3.57). Lastly, for each unit increase in cues to action, the intention to vaccinate in 3 months rather than immediately increases 1.33-fold (very small effect size - OR = 1.33, 95% CI 1.06–1.68), while the intention to vaccinate within a year rather than immediately doesn’t significantly change (very small effect size - OR = 1.27, 95% CI 0.91-1.77).

Among the incentive-related variables, only vaccine availability and receiving a Green Pass were found to be significant predictors of the sense of urgency to vaccinate 5-11-year-old children against COVID-19. Specifically, for each unit increase in vaccine availability, the intention to vaccinate in 3 months decreases 0.7-fold, while the intention to vaccinate within a year decreases 0.48-fold, compared with the intention to vaccinate immediately (very small effect size - OR = 0.70, 95% CI 0.57-0.85 and medium effect size - OR = 0.48, 95% CI 0.37-0.64, respectively). Similarly, for each unit increase in receiving a Green Pass, the intention to vaccinate in 3 months decreases 0.72-fold, while the intention to vaccinate within a year decreases 0.50-fold, compared with the intention to vaccinate immediately (very small effect size - OR = 0.72, 95% CI 0.59-0.90 and medium effect size - OR = 0.50, 95% CI 0.38-0.66, respectively). Monetary rewards and monetary penalties were not found to be significant predictors.

A complete description of the model, goodness of fit indices and regression coefficients are presented in Table 2.

**Table 2:** Multinomial logistic regression- predictors of the sense of urgency to vaccinate children aged 5–11 years once the COVID-19 vaccine is available for this age group (N = 779)

	Get vaccinated within 3 months		Get vaccinated within more than 3 months	
Variable	b(se)	*OR (95% CI]*	b(se)	*OR (95% CI]*
**Gender**				
Female	0.17(0.21)	1.19[0.78,1.81]	− 0.20(0.29)	0.82[0.46,1.45]
**Socioeconomic level**				
Low socioeconomic	− 0.35(0.27)	0.70[0.42,1.18]	− 0.40(0.35)	0.67[0.34,1.32]
High socioeconomic	− 0.39(0.25)	0.68[0.41,1.11]	− 0.41(0.34)	0.67[0.34,1.30]
**Chronic disease**				
Chronic disease in family	− 0.56*(0.25)	0.57[0.35,0.93]	− 0.39(0.33)	0.68[0.36,1.30]
**Child Flu vaccine**				
flu vaccinated	− 0.52*(0.21)	0.60[0.39,0.91]	− 0.64*(0.29)	0.53[0.30,0.92]
**HBM**				
Perceived susceptibility	− 0.45**(0.15)	0.64[0.47,0.86]	− 0.99***(0.20)	0.37[0.25,0.55]
Perceived severity	− 0.06(0.11)	0.94[0.76,1.18]	− 0.36*(0.16)	0.70[0.51,0.96]
Perceived benefits	− 0.47**(0.18)	0.62[0.44,0.89]	− 0.85***(0.23)	0.43[0.27,0.67]
Perceived barriers	0.48***(0.11)	1.62[1.30,2.02]	0.96***(0.16)	2.62[1.92,3.57]
Cues to action	0.29*(0.12)	1.33[1.06,1.68]	0.24(0.17)	1.27[0.91,1.77]
Health motivation	− 0.002(0.13)	1.00[0.77,1.29]	− 0.13(0.16)	0.88[0.65,1.21]
**Incentives**				
Availability	− 0.36***(0.10)	0.70[0.57,0.85]	− 0.73***(0.14)	0.48[0.37,0.64]
Monetary reward	− 0.05(0.07)	0.95[0.83,1.10]	− 0.17(0.11)	0.84[0.68,1.04]
Green pass	− 0.32**(0.11)	0.72[0.59,0.90]	− 0.69***(0.14)	0.50[0.38,0.66]
Monetary penalty	0.01(0.07)	1.01[0.88,1.17]	0.17(0.11)	1.18[0.96,1.46]
Constant	5.93***(1.18)		12.15***(1.55)	
$${\chi }^{2}\left(1524\right)=3389.024, p<.001$$				
$${\text{C}\text{o}\text{x} \& \text{S}\text{n}\text{e}\text{l}\text{l} R}^{2}=0.55$$				
$${\text{N}\text{a}\text{g}\text{l}\text{e}\text{k}\text{e}\text{r}\text{k}\text{e} R}^{2}=0.62$$				
$${\text{M}\text{c}\text{F}\text{a}\text{d}\text{d}\text{e}\text{n} R}^{2}=0.36$$				

*p < .05, **p < 01, ***p < .001

## Discussion and conclusion

### Discussion

This study assesses parents’ intention to vaccinate their 5-11-year-old children and determines the contributing socio-demographic, health-related and behavioral factors, as well as the role of incentives beyond these factors.

Only several studies thus far have investigated parents’ intention to vaccinate their children, with most of them conducted before COVID-19 vaccines were approved for children aged 12–16 years. The present study indicates that more than half the parents (57%) reported they will vaccinate their children in the winter of 2022 if a vaccine against COVID-19 is authorized and becomes available. This finding is consistent with the overall percentage of parents who intend to vaccinate their children (56.8%, averaged over different studies focusing on different age groups of children) according to a recent systematic review [25]. Several of the socio-demographic characteristics were found to be associated with parents’ intention to vaccinate their children were likewise in line with previous studies. For example, previous studies found that significantly higher proportions of participants who are male [26–28], older parents [25], more highly educated [27, 29] or belong to a higher income class [29, 30] exhibit a higher intention to vaccinate children against COVID-19.

The most important components of the hierarchical regression were the HBM dimensions, which added 61% to the estimate of the explained variance, on top of the 17% explained by socio-demographic and health-related characteristics. Notably, the HBM variables: perceived susceptibility and perceived benefits, displayed the largest effect sizes, where the rest of the variables presented relatively small effect sizes.

Previous studies so far have not investigated how soon parents intend to vaccinate their children against COVID-19 before the approval of the COVID-19 vaccine for this age group. This study indicates that only 27% of parents mean to vaccinate their children immediately, with a larger proportion preferring to delay the vaccination. When asked about the reasons for postponing their children’s vaccination, parents notes issues similar to those reported by previous studies, namely, concerns regarding the vaccine’s safety, serious side-effects and that the vaccine approval authorization process was carried out too quickly [29, 31, 32].

Despite this hesitancy of some of the parents, it is encouraging to see that most of the parental concerns center around the safety and efficacy of the vaccine. Such concerns can be partially addressed once the complete results of the clinical trial, which are expected to show high safety and efficacy in children, will be released to the public. Moreover, such concerns can be alleviated by publishing accessible and transparent information in the media regarding vaccine safety and efficacy as obtained from post-approval data that will accumulate rapidly as more children get vaccinated [31].

When examining the association between HBM variables and the sense of urgency to get vaccinated, perceived susceptibility and perceived barriers displayed the largest effect sizes. The association between various incentives proposed by health policy makers (i.e., monetary reward, Green Pass, etc.) and the vaccination intent was also assessed. In this study, it was found that receiving a Green Pass and administering the vaccine within the educational system are associated with increased accelerated parents’ intention to vaccinate their children, displaying medium effect sizes.

In contrast, neither monetary rewards nor monetary penalties increase parents’ intention to vaccinate their children. It should be noted, however, that the corresponding questions in the questionnaire did not mention the specific amount of the monetary reward or fine. These findings are consistent with previous studies demonstrating that monetary incentives do not increase the intention of adults to get vaccinated against COVID-19, mainly since a monetary payment for vaccination is likely to be small and is unlikely to compensate for the risk (perceived or real) of vaccination but only for the inconvenience [33, 34]. Moreover, paying people to get vaccinated offends the moral sense of individuals and the community and would have unequal effects on different segments of society [11, 35]. These concerns are expected to be magnified when it comes to vaccinating children.

Nevertheless, it is important to recognize this study’s limitations when interpreting the reported results. First, this study was conducted in Israel and, therefore, its findings might not necessarily generalize to other countries. Nevertheless, the findings of previous evaluations of vaccination intentions among adults in Israel were consistent, in general, with those conducted in other countries. For example, in [19], the author discusses such consistency in terms of the rates of intention to get vaccinated, as well as several sociodemographic predictors (e.g., age, gender, and level of education). Therefore, I believe this will be the case also with this study. Second, our participants were sampled from an online panel, an approach that inherently does not include respondents who do not have regular access to the Internet. Third, our study cohort comprised only members of the Jewish adult population in Israel and did not include members of the Arab population. Further research should be devoted to reach and include this subpopulation. Finally, it is important to be aware of the predictive limitation of a cross-sectional study. Namely, since the exposure and outcome are simultaneously assessed, it is not possible to determine a temporal or causal relationship between them.

### Conclusions

This study provides up-to-date information on the attitudes of parents to vaccinating their 5-11-year-old children, which is essential for health policy makers and healthcare providers planning vaccination campaigns. Moreover, our study’s finding that vaccine safety and side effects are key parental concerns suggests that releasing post-approval safety data regarding the vaccine to the public as soon as such information becomes available could increase parental vaccination approval rates. Finally, our findings underscore the greater effectiveness of vaccine accessibility and receiving a Green Pass, compared to other incentives, in promoting parents’ intention to vaccinate their children.

## Electronic supplementary material

Below is the link to the electronic supplementary material.

Supplementary Material 1-Supplementary Questionnaire

Supplementary Material 2

Supplementary Material 3

## Data Availability

All data generated or analysed during this study are included in this published article [and its supplementary information files].

## Footnotes

1 HBM is a socio-psychological model developed to explain and predict health-related behaviors, particularly regarding the uptake of health services. HBM has been widely used in the context of vaccination, particularly influenza vaccination [16–18] and, more recently, COVID-19 [15, 19–23].

2 These three categories were chosen based on a previous large vaccination intentions study, conducted by Ipsos in partnership with the World Economic Forum [24].

3 The socioeconomic level (before COVID-19) according to the Central Bureau of Statistics. It is based on the average gross income for a family in Israel, which is about 19,300 NIS per month when both spouses are employed and about 9,000 NIS per month for a single person, grouped into three levels: low, medium and high.

4 The ‘periphery’ level according to the Israeli Central Bureau of Statistics. It is based on a peripheral index which combines two components: potential accessibility index of the local authority and proximity of the local authority to the boundary of the Tel Aviv district (www.cbs.gov.il). The peripheral index includes local authorities that were classified into 10 clusters. I grouped those clusters based on their periphery distribution scale into three groups: peripheral, intermediate, and central.

5 According to the CDC [25] young adults, ages 18–39 have a considerably lower risk for covid-19 severe morbidity, while older adults (people aged 65 years and older) are at higher risk for severe illness from COVID-19. Since the sample in this paper only included parents of children of ages 5–11, only two individuals were of age > 65, and therefore only a single category of older ages was considered, i.e., 40+.

6 A hierarchical model was chosen so that it would be possible to assess the addition of each given layer on top of the pervious layers.

7 An invitation to fill-out the questionnaire was sent to an online panel of 5,403 participants with the goal of obtaining ~ 1,000 filled questionnaires. The invitation to fill-out the questionnaire expired after reaching 1,012 valid answers. 76 more participant started to fill-out the questionnaire but did not complete it (a completion rate of 93%).

## List of Abbreviations

FDA: The U.S. Food and Drug Administration agency
HBM: Health Belief Model
EULs: Emergency Use Listing
