# Supplementary material for: Parents’ intention to vaccinate their 5- to 11-year-old children with the COVID-19 vaccine: rates, predictors and the role of incentives
Source: BMC Public Health. 2023 Feb 14;23:328. doi: 10.1186/s12889-023-15203-y (PMC9926441; doi:10.1186/s12889-023-15203-y)
Supplement: Supplementary file 1 — Supplementary Material 1-Supplementary Questionnaire [file 12889_2023_15203_MOESM1_ESM.doc]

**Supplementary Questionnaire 1: Parental Attitudes and Intentions to Vaccinate Their 5-11-Year-Old Children with the COVID-19 Vaccine**

Approval of the vaccination against coronavirus (COVID-19) for children aged 5-11 years is expected in the coming weeks. The purpose of this study is to examine the attitudes of parents in Israel with regards to the COVID-19 vaccine for their children.

We invite you to take a few minutes to respond to a short, one-time questionnaire.

By filling out the questionnaire, you are expressing your informed consent to participate in this study and declare that you are over 18 years of age. You have the right to leave at any time without providing an explanation.

The questionnaire is anonymous and will be used for research purposes only.

Thank you for your participation in the study.

Dr. Liora Shmueli

Bar-Ilan University

1. What is your age? ___
2. Do you have children aged 5-11 years old?

1. Yes → go to question 3

2. No → Filtered from the research

1. How many children do you have according to the age range indicated in the table below?

| Number of children | Age range |
| --- | --- |
|  | 0-4 |
|  | 5-8 |
|  | 9-11 |
|  | 12-15 |
|  | 16+ |

1. Were you vaccinated against COVID-19 this year?
2. Yes, one dose
3. Yes, two doses
4. Yes, three doses
5. No, and I have no intention of getting vaccinated for COVID-19
6. Not yet, but I plan to get vaccinated for COVID-19
7. Other, detail: ____

The following items refer to your parental position with regards to a forthcoming COVID-19 vaccine for your children aged 5-11 only, if the vaccine will be made available at no cost in the upcoming winter after its approval by the FDA (the U.S. Food and Drug Administration). In each, please rate the degree to which you agree or disagree with the statement, on a 1-6 scale:

1. Strongly disagree
2. Disagree
3. Somewhat disagree
4. Somewhat agree
5. Agree
6. Strongly agree

| Strongly agree | Agree | Somewhat agree | Somewhat disagree | Disagree | Strongly disagree | Items |
| --- | --- | --- | --- | --- | --- | --- |
| 6 | 5 | 4 | 3 | 2 | 1 | 1. I intend to vaccinate my children against COVID-19 this coming winter if there is an approved vaccine |
| 6 | 5 | 4 | 3 | 2 | 1 | 1. I believe that if I my children will get vaccinated, the likelihood of them getting infected with COVID-19 will decrease |
| 6 | 5 | 4 | 3 | 2 | 1 | 1. I believe that if my children will not get vaccinated, the likelihood of our family and relatives getting infected with COVID-19 will increase |
| 6 | 5 | 4 | 3 | 2 | 1 | 1. Even if my children will get infected with COVID-19 I do not think it will cause them significant suffering or complications |
| 6 | 5 | 4 | 3 | 2 | 1 | 1. Even if my children will get infected with COVID-19, the likelihood of them recovering from the disease is very high |
| 6 | 5 | 4 | 3 | 2 | 1 | 1. I believe that the COVID-19 vaccine will be highly effective in preventing my children from experiencing significant complications of the disease |
| 6 | 5 | 4 | 3 | 2 | 1 | 1. I believe that if my children will get vaccinated against COVID-19, the likelihood that they will miss school days (school / kindergarten) will decrease |
| 6 | 5 | 4 | 3 | 2 | 1 | 1. I believe that if my children will get vaccinated against COVID-19, the likelihood of us (the parents) losing work days will decrease |
|  |  |  |  |  |  | 1. Getting vaccinated requires time and involves the loss of a workday |
| 6 | 5 | 4 | 3 | 2 | 1 | 1. The COVID-19 vaccine does not produce long-term immunity |
| 6 | 5 | 4 | 3 | 2 | 1 | 1. I am afraid that the COVID-19 vaccine has serious side effects (such as myocarditis, etc.) |
| 6 | 5 | 4 | 3 | 2 | 1 | 1. The likelihood of vaccinating my children against COVID-19 will increase if my friends and family will express support for the benefits of vaccinating children |
| 6 | 5 | 4 | 3 | 2 | 1 | 1. The likelihood of vaccinating my children against COVID-19 will increase if my pediatrician will recommend that they be vaccinated |
| 6 | 5 | 4 | 3 | 2 | 1 | 1. The likelihood of vaccinating my children against COVID-19 will increase if the vaccine will be administered by the educational system |
| 6 | 5 | 4 | 3 | 2 | 1 | 1. My children are vaccinated according to the standard immunization schedule |
|  |  |  |  |  |  |  |

1. Once the vaccine is approved and available to your 5-11-year-old children, how soon will you get them vaccinated?
2. Immediately, in less than one month→ Go to question 22
3. Within 1-3 months→ Go to question 21
4. Within 4-6 months→ Go to question 21
5. Within 7-12 months→ Go to question 21
6. After 12 months→ Go to question 21
7. I do not intend to vaccinate my children at all→ Go to question 21
8. What are the reasons for which you do not intend to vaccinate your children immediately/at all as soon as the vaccine is made available to them?

(Shown only to those who chose options 2-6 in question 20, more than one reason may be selected)

1. I am afraid that the clinical trials and their approval process were conducted too quickly, for political reasons
2. I have concerns about the vaccine’s safety
3. I have concerns about the vaccine’s low effectiveness
4. I am afraid the vaccine will have severe side effects
5. I think that Covid-19 is not dangerous to children, and therefore there is no reason to vaccinate them
6. If all the adults in my environment are vaccinated, there is no need to vaccinate my children
7. My children fear vaccines and injections
8. I am against all types of vaccinations in general
9. Other, detail: ________

**Incentives**

Please indicate the degree to which you think each of the following items will increase your intention to vaccinate your children for COVID-19, on a 1-6 scale:

1. Will not increase at all
2. Will not increase
3. Will not increase significantly
4. Will increase somewhat
5. Will increase
6. Will increase considerably

|  | | | | | Will increase considerably | | Will increase | | Will increase somewhat | Will not increase significantly | Will not increase | Will not increase at all |  |
| --- | --- | --- | --- | --- | --- | --- | --- | --- | --- | --- | --- | --- | --- |
| 6 | 5 | 4 | 3 | 2 | | 1 | | 1. If the vaccine would become accessible and available at school | | | | | |
| 6 | 5 | 4 | 3 | 2 | | 1 | | 1. If I would receive a monetary reward | | | | | |
| 6 | 5 | 4 | 3 | 2 | | 1 | | 1. If my child will receive a ‘Green Pass’ granting various exemptions (exemption from isolation, boarding flights without prior COVID testing, entry to entertainment venues, etc.) | | | | | |
| 6 | 5 | 4 | 3 | 2 | | 1 | | 1. If I will be fined (e.g., the government would reduce my social security benefits or impose another fine in the case that my child does not get vaccinated) | | | | | |

|  | In each of the following items, please indicate the degree to which you agree or disagree with the statement, on a 1-6 scale. | | | | | | | |
| --- | --- | --- | --- | --- | --- | --- | --- | --- |
| Strongly agree | | Agree | Somewhat agree | Somewhat disagree | Disagree | Strongly disagree |  |  |
| 6 | | 5 | 4 | 3 | 2 | 1 | 1. I intend to have my children vaccinated against the flu virus this winter |  |

1. Did your children receive a seasonal flu vaccine last winter?
2. Yes
3. No

**Additional questions regarding your demographic background**

1. Are you a medical staff member who treats or sees patients (including nursing homes, etc.)?
2. Yes
3. No
4. Do you, or does a family member living at home with you, suffer from a health/chronic problem as a result of one or more of the following underlying diseases:
5. Heart disease, vascular disease and/or stroke
6. Diabetes mellitus
7. Hypertension
8. Chronic lung disease, including asthma (not including childhood asthma)
9. Renal failure
10. Immunosuppression including taking immune-suppressing or cancer medications
11. No family member suffers from any of the above underlying diseases
12. Were you, or was a family member living at home with you, diagnosed with COVID-19 in the past?
13. Yes
14. No
15. I don't know
16. Decline to answer
17. Were you, or was a family member, hospitalized in the past 12 months? Mark one or more as relevant:
18. Yes, due to flu complications
19. Yes, due to COVID-19 symptoms/complications
20. Yes, for another reason
21. No
22. Decline to answer

The following question is relevant to those who responded to question no. 3 above that they have children 12-15 years old.

1. Did your children 12-15 years old receive a vaccination for COVID-19?
2. Yes
3. No
4. Year of birth: _____
5. Respondent’s gender: (1) Male (2) Female (3) Other
6. What is the highest level of education you have completed?
7. Elementary
8. High school
9. High school - non-academic
10. Academic – bachelor's degree
11. Academic – master's degree or higher
12. Marital status
13. Single
14. Married/in a relationship
15. Divorced/separated
16. Widowed
17. What is your religion?
18. Jewish
19. Muslim
20. Christian
21. Druze
22. Other, specify: ____________
23. Degree of religiosity
24. Secular
25. Traditional
26. Religious
27. Orthodox
28. Socio-economic level (prior to the COVID-19 period). According to Israel’s Central Bureau of Statistics, the average gross montly income for a household in Israel is NIS 19,300 when both spouses are employed (NIS 9,000 for a single income-earner). How would you rate your income?
29. Significantly below average
30. Slightly below average
31. Average
32. Slightly above average\
33. Significantly above average
34. Town of residence: ________

Thank you for participating in the study.
