# Supplementary material for: Parents’ intention to vaccinate their 5- to 11-year-old children with the COVID-19 vaccine: rates, predictors and the role of incentives
Source: BMC Public Health. 2023 Feb 14;23:328. doi: 10.1186/s12889-023-15203-y (PMC9926441; doi:10.1186/s12889-023-15203-y)
Supplement: Supplementary file 2 — Supplementary Material 2 [file 12889_2023_15203_MOESM2_ESM.docx]

**Supplementary Tables**

**Supplementary Table 1. Items and internal consistency for assessing measures of the HBM**

| Measures | Items | a |
| --- | --- | --- |
| Perceived susceptibility | I believe that if I my children will get vaccinated, the likelihood of them getting infected with COVID-19 will decrease | 0.87 |
|  | I believe that if my children will not get vaccinated, the likelihood of our family and relatives getting infected with COVID-19 will increase |  |
| *Perceived severity | Even if my children will get infected with COVID-19 I do not think it will cause them significant suffering or complications | 0.63 |
|  | Even if my children will get infected with COVID-19, the likelihood of them recovering from the disease is very high |  |
| Perceived Benefits | I believe that the COVID-19 vaccine will be highly effective in preventing my children from experiencing significant complications of the disease | 0.89 |
|  | I believe that if my children will get vaccinated against COVID-19, the likelihood that they will miss school days (school / kindergarten) will decrease |  |
|  | I believe that if my children will get vaccinated against COVID-19, the likelihood of us (the parents) losing work days will decrease |  |
| Perceived barriers | Getting vaccinated requires time and involves the loss of a workday | 0.68 |
|  | The COVID-19 vaccine does not produce long-term immunity |  |
|  | I am afraid that the COVID-19 vaccine has serious side effects (such as myocarditis etc.) |  |
| Cues to action | The likelihood of vaccinating my children against COVID-19 will increase if my friends and family will express their support for the benefits of vaccinating children | 0.84 |
|  | The likelihood of vaccinating my children against COVID-19 will increase if my pediatrician will recommend that they be vaccinated |  |
|  | The likelihood of vaccinating my children against COVID-19 will increase if the vaccine will be administered by the educational system |  |
| Health motivation | My children are vaccinated according to the standard immunization schedule | - |

a Cronbach indicates the internal consistency: **HBM** a= 0.79

Items response scale: 1-6 agreement

* Negative items were reverse scored

**Supplementary Table 2: Comparing the sample and the entire population in terms of the characteristics used for sampling: age, gender, level of religiosity, and geographical area**

| **Sociodemographic** | **Population** | **sample** | |
| --- | --- | --- | --- |
|  |  | **(n=1012)** | |
|  | % | N | % |
| **Age group *** |  |  |  |
| 18-39 | 50% | 489 | 48% |
| 40-65 | 50% | 523 | 52% |
| **Gender *** |  |  |  |
| Male | 50% | 496 | 49% |
| Female | 50% | 516 | 51% |
| **Level of religiosity **** |  |  |  |
| Secular | 45% | 498 | 50% |
| Traditional | 25% | 284 | 28% |
| Religious | 16% | 122 | 12% |
| Haredi | 14% | 103 | 10% |
| **Geographical area **** |  |  |  |
| *Jerusalem District* | 11% | 76 | 8% |
| *Northern District* | 9% | 121 | 12% |
| *Haifa District* | 11% | 139 | 14% |
| *Central District* | 29% | 305 | 30% |
| *Tel Aviv District* | 20% | 167 | 17% |
| *Southern District* | 14% | 156 | 15% |
| *Judea and Samaria Area* | 6% | 48 | 5% |

* Jewish population, ages 18-65 (n=3.5 million)

** Jewish population, all ages (n=6.4 million)

**Supplementary Table 3: Characteristics of respondents by the intention to vaccinate their children against COVID-19**

| **Sociodemographic** | **All subjects**  **(n=1,012)** | | ***DO not intend to vaccinate children against COVID-19**  **N=433 (42.8%)** | | ***Intend to vaccinate children against COVID-19**  **N=579 (57.2%)** | | $\boldsymbol{\chi}\mathbf{2}$ | **p-value** |
| --- | --- | --- | --- | --- | --- | --- | --- | --- |
|  | N | (%) | N | (%) | N | (%) |  |  |
| **Age group** |  |  |  |  |  |  |  |  |
| 18-39 | 489 | (48.3%) | 248 | (50.7%) | 241 | (49.3%) | $\chi2(2)=24.30$ | <0.001 |
| 40+ | 523 | (51.7%) | 185 | (35.4%) | 338 | (64.4%) |  |  |
| **Gender**  Male | 496 | (49%) | 184 | (37.1%) | 312 | (62.9%) | $\chi2(1)=12.87$ | <0.001 |
| Female | 516 | (51%) | 249 | (48.3%) | 267 | (51.7%) |  |  |
| **Education level**  High school or less | 142 | (14%) | 68 | (47.9%) | 74 | (52.1%) | $\chi2(3)=10.84$ | 0.013 |
| Non-academic | 245 | (24.2%) | 115 | (46.9%) | 130 | (53.1%) |  |  |
| BA | 418 | (41.3%) | 181 | (43.3%) | 237 | (56.7%) |  |  |
| MA or higher | 207 | (20.5%) | 69 | (33.3%) | 138 | (66.7%) |  |  |
| **Personal status**  Single | 32 | (3.2%) | 18 | (56.3%) | 14 | (43.8%) | $\chi2(3)=3.65$ | 0.301 |
| Married | 902 | (89.1%) | 386 | (42.8%) | 516 | (57.2%) |  |  |
| Divorced | 74 | (7.3%) | 27 | (36.5%) | 47 | (63.5%) |  |  |
| Widow | 4 | (0.4%) | 2 | (50%) | 2 | (50%) |  |  |
| **Socio-economic level** | | |  |  |  |  | $\chi2(2)=19.52$ |  |
| Low | 336 | (33.2%) | 160 | (47.6%) | 176 | (52.4%) |  | <0.001 |
| Medium | 345 | (34.1%) | 164 | (47.5%) | 181 | (52.5%) |  |  |
| High | 331 | (32.7%) | 109 | (32.9%) | 222 | (67.1%) |  |  |
| **Peripheral level**  Periphery | 135 | (13.4%) | 58 | (43%) | 77 | (57%) |  | 0.96 |
| Intermediate | 439 | (43.5%) | 186 | (42.4%) | 253 | (57.6%) | $\chi2\left( 2 \right)=.09$ |  |
| Central | 436 | (43.2%) | 189 | (43.3%) | 247 | (56.7%) |  |  |
| **Medical Staff** |  |  |  |  |  |  |  |  |
| No | 963 | (95.2%) | 411 | (42.7%) | 552 | (57.3%) | $\chi2\left( 1 \right)=.09$ | .76 |
| Yes | 49 | (4.8%) | 22 | (44.9%) | 27 | (55.1%) |  |  |
| **Religiosity** |  |  |  |  |  |  |  |  |
| Secular | 498 | (49.5%) | 194 | (39%) | 304 | (61%) | $\chi2\left( 3 \right)=7.32$ | .062 |
| Traditional | 284 | (28.2%) | 138 | (48.6%) | 146 | (51.4%) |  |  |
| Religious | 122 | (12.1%) | 55 | (45.1%) | 67 | (54.9%) |  |  |
| Haredi | 103 | (10.2%) | 42 | (40.8%) | 61 | (59.2%) |  |  |

| **Health related variables** | | **All subjects**  **(n=1,012)** | | | **DO not intend to vaccinate children against COVID-19**  **N=433 (42.8%)** | | | **Intend to vaccinate children against COVID-19**  **N=579 (57.2%)** | | $\boldsymbol{\chi}\mathbf{2}$ | **p-value** |
| --- | --- | --- | --- | --- | --- | --- | --- | --- | --- | --- | --- |
|  | N | | | (%) | N | (%) | N | | (%) |  |  |
| **Chronic Disease**  No chronic disease | | | 781 | (77.2%) | 342 | (43.8%) | 439 | | (56.2%) | $\chi2\left( 1 \right)=1.41$ | .24 |
| Chronic disease | | | 231 | (22.8%) | 91 | (39.4%) | 140 | | (60.6%) |  |  |
| **Past episodes of COVID-19**  No | | | 704 | (70.8%) | 296 | (42%) | 408 | | (58%) | $\chi2(1)=0.17$ | .68 |
| Yes | | | 290 | (29.2%) | 126 | (43.4%) | 164 | | (56.6%) |  |  |
| **Flu vaccine last year (kids)** | | |  |  |  |  |  | |  | $\chi2\left( 1 \right)=34.88$ | <.001 |
| No | | | 539 | (53.3%) | 277 | (51.4%) | 262 | | (48.6%) |  |  |
| Yes | | | 473 | (46.7%) | 156 | (33%) | 317 | | (67%) |  |  |
| **A vaccinated parent to COVID-19** | | |  |  |  |  |  | |  | $\chi2\left( 1 \right)=72.81$ | <.001 |
| No | | | 89 | (8.8%) | 76 | (85.4%) | 13 | | (14.6%) |  |  |
| Yes | | | 921 | (91.2%) | 355 | (38.5%) | 566 | | (61.5%) |  |  |
| **Past episodes of hospitalization** | | | | |  |  |  | |  | $\chi2\left( 1 \right)=.43$ | .51 |
| No | | | 919 | (91.8%) | 393 | (42.8%) | 526 | | (57.2%) |  |  |
| Yes | | | 82 | (8.2%) | 32 | (39%) | 50 | | (61%) |  |  |
| **Kids 12-15 vaccinated to COVID-19 (n=422)** | | | | |  |  |  | |  | $\chi2\left( 1 \right)=42.82$ | <.001 |
| No | | | 208 | (49.3%) | 112 | (53.8%) | 96 | | (46.2%) |  |  |
| Yes | | | 214 | (50.7%) | 49 | (22.9%) | 165 | | (77.1%) |  |  |

Notes:

*Parents’ intention to vaccinate their 5-11-year-old children against COVID-19 in the winter of 2022, was originally measured as a one-item question on a 1-6 scale (1 - strongly disagree; 6 - strongly agree). This variable was transformed to a binary variable (1 – I intend to vaccinate my children; 0 – I do not intend to vaccinate my children).

**Percentages of “Do not Intend to get children vaccinated against COVID-19” and “Intend to get children vaccinated against COVID-19” are calculated as valid % per each row (i.e., each row sums up to 100%, without missing values).

**Supplementary Table 4**: Univariate analyses between HBM, incentives variables and willingness to vaccinate your children against COVID-19

| **Variables** | DO not-intend to vaccinate children  (n= 433)  Mean (SD) | | | Intend to get vaccinate children  (n= 579)  Mean (SD) | | | | t-test | | | P value (two-tail) | | | Effect  Size  Cohen's d |
| --- | --- | --- | --- | --- | --- | --- | --- | --- | --- | --- | --- | --- | --- | --- |
| **HBM variables** |  | | | |  |  | | |  |  | |  |  | |
| Perceived Susceptibility | 2.77 | (1.20) | 4.98 | | | (.91) | -33.03 | | | | <.001 | | 2.08 | |
| Perceived Severity | 2.52 | (1.10) | 2.55 | | | (.96) | -.48 | | | | .63 | | 0.03 | |
| Perceived Benefits | 3.19 | (1.20) | 5.07 | | | (.81) | -28.18 | | | | <.001 | | 1.83 | |
| Perceived Barriers | 4.98 | (1.01) | 3.78 | | | (1.00) | 18.78 | | | | <.001 | | 1.19 | |
| Cues to action | 2.46 | (1.14) | 4.14 | | | (1.07) | -23.79 | | | | <.001 | | 1.52 | |
| Health motivation | 4.86 | (1.41) | 5.37 | | | (.93) | -6.60 | | | | <.001 | | 0.43 | |
| **Incentives variables** |  |  |  | | |  |  | | | |  | |  | |
| Availability | 2.05 | (1.20) | 4.60 | | | (1.34) | -31.82 | | | | <.001 | | 2.00 | |
| Monetary reward | 1.94 | (1.28) | 3.89 | | | (1.81) | -20.02 | | | | <.001 | | 1.24 | |
| Green pass | 2.43 | (1.47) | 4.94 | | | (1.27) | -28.59 | | | | <.001 | | 1.83 | |
| Monetary penalty | 2.22 | (1.40) | 3.94 | | | (1.72) | -17.5 | | | | <.001 | | 1.09 | |
|  |  | | | |  |  | | |  |  | |  |  | |

Note: COVID-19 vaccination intention measured by the item: “I want to vaccinate my children against the COVID-19 virus as soon as a vaccine is available”, on a 1-6 agreement scale

HBM and incentives Items Response scale: 1-6 agreement

**Supplementary Table 5: Characteristics of respondents by sense of urgency to vaccinate their children against COVID-19**

| **Sociodemographic** | **All subjects** | | **Immediately** | | **Within 3 months** | | **More than 3 months** | | $\boldsymbol{\chi}\mathbf{2}$ | **p-value** | |
| --- | --- | --- | --- | --- | --- | --- | --- | --- | --- | --- | --- |
|  | N=779 | (%) | N=270 | (34.7%) | N=267 | (34.3%) | N=242 | (31.1%) |  |  |  |
| **Age group** |  |  |  |  |  |  |  |  | $\chi2\left( 2 \right)=4.76$ | .09 | |
| 18-39 | 348 | (44.7%) | 108 | (31%) | 120 | (34.5%) | 120 | (34.5%) |  |  | |
| 40+ | 431 | (55.3%) | 162 | (37.6%) | 147 | )34.1%) | 122 | (28.3%) |  |  | |
| **Gender**  Male | 398 | (51.1%) | 157 | (39.4%) | 126 | (31.7%) | 115 | (28.9%) | $\chi2(2)=8.24$ | 0.016 | |
| Female | 381 | (48.9%) | 113 | (29.7%) | 141 | (37%) | 127 | (33.3%) |  |  | |
| **Educational level**  High school or less | 98 | (12.6%) | 39 | (39.8%) | 33 | (33.7%) | 26 | (26.5%) | $\chi2(6)=3.63$ | 0.726 | |
| Non-academic | 182 | (23.4%) | 62 | (34.1%) | 65 | (35.7%) | 55 | (30.2%) |  |  | |
| BA | 325 | (41.7%) | 104 | (32%) | 111 | (34.2%) | 110 | (33.8%) |  |  | |
| MA or higher | 174 | (22.3%) | 65 | (37.4%) | 58 | (33.3%) | 51 | (29.3%) |  |  | |
| **Personal status-partnership** | | |  |  |  |  |  |  | $\chi2\left( 6 \right)=4.17$ | 0.653 | |
| Single | 21 | (2.7%) | 6 | (28.6%) | 6 | (28.6%) | 9 | (42.9%) |  |  | |
| Married | 696 | (89.3%) | 240 | (34.5%) | 239 | (34.3%) | 696 | (31.2%) |  |  | |
| Divorced | 59 | (7.6%) | 22 | (37.3%) | 22 | (37.3%) | 59 | (25.4%) |  |  | |
| Widow | 3 | (0.4%) | 2 | (66.7%) | 0 | (0%) | 1 | (33.3%) |  |  | |
| **Socioeconomic level** | | |  |  |  |  |  |  | $\chi2(4)=11.31$ | 0.023 | |
| Low | 237 | (30.4%) | 81 | (34.2%) | 84 | (35.4%) | 72 | (30.4%) |  |  | |
| Middle | 256 | (32.9%) | 71 | (27.7%) | 94 | (36.7%) | 91 | (35.5%) |  |  | |
| High | 286 | (36.7%) | 118 | (41.3%) | 89 | (31.1%) | 79 | (27.6%) |  |  | |
| **Peripheral level** |  |  |  |  |  |  |  |  | $\chi2\left( 4 \right)=1.36$ | 0.851 | |
| Periphery | 108 | (13.9%) | 37 | (34.3%) | 34 | (31.5%) | 37 | (34.3%) |  |  | |
| Intermediate | 347 | (44.7%) | 123 | (35.4%) | 116 | (33.4%) | 108 | (31.1%) |  |  | |
| Central | 322 | (41.4%) | 109 | (33.9%) | 117 | (36.3%) | 96 | (29.8%) |  |  | |
| **Medical Staff** |  |  |  |  |  |  |  |  | $\chi2\left( 2 \right)=5.37$ | .068 | |
| No | 747 | (95.9%) | 256 | (34%) | 253 | (33.9%) | 238 | (31.9%) |  |  | |
| Yes | 32 | (4.1%) | 14 | (43.8%) | 14 | (43.8%) | 4 | (12.5%) |  |  | |
| **Religiosity** |  |  |  |  |  |  |  |  | $\chi2\left( 6 \right)=6.40$ | .38 | |
| Secular | 404 | (52%) | 147 | (36.4%) | 134 | (33.2%) | 123 | (30.4%) |  |  | |
| Traditional | 208 | (26.8%) | 63 | (30.3%) | 74 | (35.6%) | 71 | (34.1%) |  |  | |
| Religious | 93 | (12%) | 30 | (32.3%) | 32 | (34.4%) | 31 | (33.3%) |  |  | |
| Haredi | 72 | (9.3%) | 30 | (41.7%) | 27 | (37.5%) | 15 | (20.8%) |  |  | |

| **Health related variables** | **All subjects** | | | **Immediately** | | **Within 3 months** | | **More than 3 months** | | $\boldsymbol{\chi}\mathbf{2}$ | **p-value** |
| --- | --- | --- | --- | --- | --- | --- | --- | --- | --- | --- | --- |
|  | | N | (%) | N | (%) | N | (%) | N | (%) |  |  |
| **Chronic Disease**  No chronic disease | | 600 | (77%) | 195 | (32.5%) | 219 | (36.5%) | 186 | (31%) | $\chi2\left( 2 \right)=7.29$ | .026 |
| Chronic disease | | 179 | (23%) | 75 | (41.9%) | 48 | (26.8%) | 56 | (31.3%) |  |  |
| **Past episodes of COVID-19**  No | | 562 | (73.4%) | 192 | (34.2%) | 195 | (34.7%) | 175 | (31.1%) | $\chi2\left( 2 \right)=.47$ | .791 |
| Yes | | 204 | (26.6%) | 75 | (36.8%) | 67 | (32.8%) | 62 | (30.4%) |  |  |
| **Flu vaccine last year (kids)** | | | |  |  |  |  |  |  | $\chi2\left( 2 \right)=21.99$ | <.001 |
| No | | 382 | (49%) | 104 | (27.2%) | 135 | (35.3%) | 143 | (37.4%) |  |  |
| Yes | | 397 | (51%) | 166 | (41.8%) | 132 | (33.2%) | 99 | (24.9%) |  |  |
| **A vaccinated parent to COVID-19** | | | |  |  |  |  |  |  | $\chi2\left( 2 \right)=2.28$ | .32 |
| No | | 17 | (2.2%) | 3 | (17.6%) | 7 | (41.2%) | 7 | (41.2%) |  |  |
| Yes | | 761 | (97.8%) | 267 | (35.1%) | 260 | (34.2%) | 234 | (30.7%) |  |  |
| **Past episodes of hospitalization** | | | |  |  |  |  |  |  | $\chi2\left( 2 \right)=.84$ | .66 |
| No | | 705 | (91.2%) | 246 | (34.9%) | 238 | (33.8%) | 221 | (31.3%) |  |  |
| Yes | | 68 | (8.8%) | 24 | (35.3%) | 26 | (38.2%) | 18 | (26.5%) |  |  |
| **Kids 12-15 vaccinated with COVID-19 (n=422)** | | | |  |  |  |  |  |  | $\chi2\left( 2 \right)=2.21$ | .33 |
| No | | 130 | (39%) | 47 | (36.2%) | 42 | (32.3%) | 41 | (31.5%) |  |  |
| Yes | | 203 | (61%) | 82 | (40.4%) | 72 | (35.5%) | 49 | (24.1%) |  |  |

Note: Percentages of “Do not Intend to get vaccinated against COVID-19” and “Intend to get vaccinated against COVID-19” are calculated as valid % per each row (i.e., each row sums up to 100%, without missing values).

**p*<0.05

**Supplementary Table 6:** Univariate analyses between HBM, incentives variables and the sense of urgency to vaccinate children against COVID-19

|  | **Immediately**  **(n= 270)** | | **Within 3 months (n= 267)** | | **More than 3 months**  **(n= 242)** | | **F-test** | **P value** | $\boldsymbol{\eta}^{\boldsymbol{2}}$ |
| --- | --- | --- | --- | --- | --- | --- | --- | --- | --- |
| **Variables** | Mean | (SD) | Mean | (SD) | Mean | (SD) |  |  |  |
| **HBM variables** |  |  |  |  |  |  |  |  |  |
| Perceived Susceptibility | 5.34 | (.79) | 4.66 | (.91) | 3.62 | (1.03) | 226.67 | <.001 | .37 |
| Perceived Severity | 2.50 | (1.00) | 2.63 | (.94) | 2.62 | (.92) | 1.47 | .232 | 0.00 |
| Perceived Benefits | 5.39 | (.71) | 4.79 | (.83) | 3.90 | (.95) | 203.86 | <.001 | 0.34 |
| Perceived Barriers | 3.43 | (1.05) | 4.05 | (.86) | 4.63 | (.93) | 103.36 | <.001 | 0.21 |
| Cues to action | 4.23 | (1.18) | 4.08 | (.93) | 3.27 | (1.04) | 60.27 | <.001 | 0.13 |
| Health motivation | 5.54 | (.81) | 5.29 | (.95) | 4.99 | (1.17) | 20.23 | <.001 | 0.05 |
| **Incentives variables** |  |  |  |  |  |  |  |  |  |
| Availability | 5.11 | (1.21) | 4.25 | (1.27) | 2.82 | (1.25) | 220.73 | <.001 | 0.36 |
| Monetary reward | 4.33 | (1.84) | 3.65 | (1.70) | 2.45 | (1.43) | 82.36 | <.001 | 0.18 |
| Green pass | 5.42 | (1.04) | 4.67 | (1.30) | 3.24 | (1.36) | 201.30 | <.001 | 0.34 |
| Monetary penalty | 4.30 | (1.80) | 3.68 | (1.59) | 2.86 | (1.45) | 50.32 | <.001 | 0.12 |

Note: the sense of urgency to receive COVID-19 vaccine measured by the item: "now as the vaccine is available, how soon will you get you children vaccinated? Immediately, within 3 months or within a year?".

HBM and incentives Items Response scale: 1-6 agreement

|  | **Model 1** | | | | | **Model 2** | | | | | | **Model 3** | | | | | | **Model 4** | | | | |
| --- | --- | --- | --- | --- | --- | --- | --- | --- | --- | --- | --- | --- | --- | --- | --- | --- | --- | --- | --- | --- | --- | --- |
| *Predictors* | *b* | *se* | β | *p* | *95%CI* | *b* | *se* | β | *p* | *95%CI* | *b* | | *se* | β | *p* | *95%CI* | *b* | | *se* | β | *p* | *95%CI* |
| **Socio-demographic** |  |  |  |  |  |  |  |  |  |  |  | |  |  |  |  |  | |  |  |  |  |
| Age Group | 0.45 | 0.11 | 0.13 | **<0.001** | 0.24 – 0.66 | 0.37 | 0.1 | 0.11 | **<0.001** | 0.17 – 0.57 | 0.22 | | 0.06 | 0.06 | **<0.001** | 0.11 – 0.33 | 0.18 | | 0.05 | 0.05 | **0.001** | 0.07 – 0.28 |
| Gender (Female) | -0.36 | 0.11 | -0.11 | **0.001** | -0.58 – -0.16 | -0.4 | 0.1 | -0.12 | **<0.001** | -0.60 – -0.21 | -0.06 | | 0.06 | -0.02 | 0.331 | -0.17 – 0.06 | -0.02 | | 0.05 | -0.01 | 0.675 | -0.13 – 0.08 |
| Education (Academaic) | 0.09 | 0.11 | 0.03 | 0.41 | -0.12 – 0.34 | -0.03 | 0.11 | -0.01 | 0.788 | -0.24 – 0.18 | -0.03 | | 0.06 | -0.01 | 0.665 | -0.15 – 0.09 | -0.02 | | 0.06 | 0 | 0.784 | -0.12 – 0.09 |
| Low SES | 0.04 | 0.12 | 0.01 | 0.76 | -0.20 – 0.32 | 0.05 | 0.12 | 0.01 | 0.676 | -0.19 – 0.30 | 0.04 | | 0.07 | 0.01 | 0.525 | -0.09 – 0.18 | 0.05 | | 0.06 | 0.01 | 0.432 | -0.08 – 0.18 |
| High SES | 0.52 | 0.13 | 0.14 | **<0.001** | 0.26 – 0.77 | 0.42 | 0.12 | 0.11 | **0.001** | 0.18 – 0.66 | 0.08 | | 0.07 | 0.02 | 0.249 | -0.06 – 0.22 | 0.11 | | 0.06 | 0.03 | 0.095 | -0.02 – 0.23 |
| **Health-related** |  |  |  |  |  |  |  |  |  |  |  | |  |  |  |  |  | |  |  |  |  |
| Vaccine parent (COVID-19) |  |  |  |  |  | 1.74 | 0.18 | 0.29 | **<0.001** | 1.39 – 2.10 | -0.22 | | 0.11 | -0.04 | **0.047** | -0.44 – -0.00 | -0.25 | | 0.1 | -0.04 | **0.013** | -0.45 – -0.05 |
| Vaccine child (Flu) |  |  |  |  |  | 0.53 | 0.1 | 0.15 | **<0.001** | 0.33 – 0.73 | 0.04 | | 0.06 | 0.01 | 0.491 | -0.08 – 0.16 | 0.05 | | 0.05 | 0.01 | 0.342 | -0.05 – 0.16 |
| **HBM** |  |  |  |  |  |  |  |  |  |  |  | |  |  |  |  |  | |  |  |  |  |
| Perceived Susceptibility |  |  |  |  |  |  |  |  |  |  | 0.5 | | 0.04 | 0.43 | **<0.001** | 0.43 – 0.57 | 0.4 | | 0.03 | 0.35 | **<0.001** | 0.33 – 0.46 |
| Perceived Severity |  |  |  |  |  |  |  |  |  |  | 0.02 | | 0.03 | 0.01 | 0.427 | -0.03 – 0.08 | 0.03 | | 0.03 | 0.02 | 0.204 | -0.02 – 0.08 |
| Perceived Benefits |  |  |  |  |  |  |  |  |  |  | 0.28 | | 0.04 | 0.22 | **<0.001** | 0.20 – 0.35 | 0.19 | | 0.04 | 0.15 | **<0.001** | 0.12 – 0.26 |
| Perceived Barriers |  |  |  |  |  |  |  |  |  |  | -0.26 | | 0.03 | -0.17 | **<0.001** | -0.32 – -0.20 | -0.17 | | 0.03 | -0.12 | **<0.001** | -0.23 – -0.12 |
| Perceived Cues to action |  |  |  |  |  |  |  |  |  |  | 0.22 | | 0.03 | 0.18 | **<0.001** | 0.17 – 0.28 | 0.03 | | 0.03 | 0.02 | 0.285 | -0.03 – 0.09 |
| Perceived Health motivation |  |  |  |  |  |  |  |  |  |  | -0.01 | | 0.03 | -0.01 | 0.688 | -0.06 – 0.04 | 0 | | 0.02 | 0 | 0.958 | -0.05 – 0.05 |
| **Incentives** |  |  |  |  |  |  |  |  |  |  |  | |  |  |  |  |  | |  |  |  |  |
| Availability |  |  |  |  |  |  |  |  |  |  |  | |  |  |  |  | 0.25 | | 0.03 | 0.26 | **<0.001** | 0.20 – 0.31 |
| Monetary reward |  |  |  |  |  |  |  |  |  |  |  | |  |  |  |  | -0.02 | | 0.02 | -0.02 | 0.449 | -0.06 – 0.03 |
| Green pass |  |  |  |  |  |  |  |  |  |  |  | |  |  |  |  | 0.15 | | 0.03 | 0.16 | **<0.001** | 0.09 – 0.20 |
| Monetary penalty |  |  |  |  |  |  |  |  |  |  |  | |  |  |  |  | -0.02 | | 0.02 | -0.02 | 0.396 | -0.06 – 0.02 |
| (Intercept) | 3.48 | 0.14 | 0 | **<0.001** | 3.20 – 3.76 | 1.81 | 0.2 | 0 | **<0.001** | 1.42 – 2.20 | 0.99 | | 0.25 | 0 | **<0.001** | 0.49 – 1.48 | 0.63 | | 0.23 | 0 | **0.006** | 0.18 – 1.09 |
| Observations | 1012 | | | | | 1012 | | | | | | 1012 | | | | | | 1012 | | | | |
| R^2^ | 0.06 | | | | | 0.174 | | | | | | 0.742 | | | | | | 0.788 | | | | |
| F | 12.52, p < .001 | | | | | 30.24, p < .001 | | | | | | 220.64, p < .001 | | | | | | 216.27, p < .001 | | | | |
| ΔR2 |  | | | | | 0.12 | | | | | | 0.57 | | | | | | 0.05 | | | | |
| ΔF |  | | | | | 70.23, p < .001 | | | | | | 365.72, p < .001 | | | | | | 52.83, p < .001 | | | | |

**Supplementary Table 7:** Hierarchical linear regression analysis - predictors of parents’ intention to vaccinate their children aged 5-11 years against COVID-19 (n=1,012)
