# Supplementary material for: Parents’ intention to vaccinate their 5- to 11-year-old children with the COVID-19 vaccine: rates, predictors and the role of incentives
Source: BMC Public Health. 2023 Feb 14;23:328. doi: 10.1186/s12889-023-15203-y (PMC9926441; doi:10.1186/s12889-023-15203-y)
Supplement: Supplementary file 3 — Supplementary Material 3 [file 12889_2023_15203_MOESM3_ESM.doc]

**Supplementary Analysis 1: Hierarchical linear regression analysis - predictors of parents’ intention to vaccinate their children aged 5-11 years against COVID-19**

For robustness, an alternative analysis was conducted in which the intention to vaccinate variable was analyzed in its original scale (without transformation). In this analysis, a hierarchical linear regression was conducted, with four blocks of variables, identical to those used in the hierarchical logistic regression analysis, where the target variable - the intention to vaccinate children – was kept without transformation.

The results of the linear regression analysis are reported in Supplementary Table 7. The results are consistent with those obtained in the logistic regression analysis. Specifically, the model accounted for an estimated 79% (compared to 80% in the logistic regression analysis) of the explained variance in the intention of 5-11-year-old children to vaccinate against COVID-19 in the winter of 2022 (adjusted R square = 0.79). All the model’s steps were significant. The most important components of the linear regression analysis were the HBM dimensions, which added 57% (compared to 61% in the logistic regression analysis) to the estimate of the explained variance, on top of the 17% (compared to 17% in the logistic regression analysis) explained by socio-demographic and health-related characteristics.
